# A Plasma Membrane Intrinsic Protein Gene *OfPIP2* Involved in Promoting Petal Expansion and Drought Resistance in *Osmanthus fragrans*

**DOI:** 10.3390/ijms251910716

**Published:** 2024-10-05

**Authors:** Xinke Lu, En Kong, Lixiao Shen, Yong Ye, Yiguang Wang, Bin Dong, Shiwei Zhong

**Affiliations:** 1 School of Landscape Architecture, Zhejiang Agriculture and Forestry University, Hangzhou 311300, China; 19858167122@163.com (X.L.); 19157843156@163.com (E.K.); shenlixiao@stu.zafu.edu.cn (L.S.); 2021105051003@stu.zafu.edu.cn (Y.Y.); wangyiguang1990@163.com (Y.W.); 2 Zhejiang Provincial Key Laboratory of Germplasm Innovation and Utilization for Garden Plants, Hangzhou 311300, China

**Keywords:** aquaporin, flowering, drought stress, *Osmanthus fragrans*

## Abstract

*Osmanthus fragrans*, a native to China, is renowned as a highly popular gardening plant. However, this plant faces significant challenges from drought stress, which can adversely affect its flowering. In this study, we found that the plasma membrane-localized gene *OfPIP2* exhibited a substantial upregulation during the flowering stages and in response to drought stress. GUS staining has illustrated that the *OfPIP2* promoter can drive GUS activity under drought conditions. The overexpression of *OfPIP2* was found to enhance petal size by modulating epidermal cell dimensions in *Petunia* and tobacco. Moreover, this overexpression also bolstered drought tolerance, as evidenced by a reduction in stomatal aperture in both species. Furthermore, yeast one-hybrid (Y1H) and dual-luciferase (Dual-LUC) assays have indicated that the transcription factor OfMYB28 directly binds to the *OfPIP2* promoter, thereby regulating its expression. Together, we speculated that a module of OfMYB28-*OfPIP2* was not only involved in the enhancement of petal size but also conferred the improvement of drought tolerance in *O. fragrans*. These results contribute valuable insights into the molecular function of the *OfPIP2* gene and lay a foundation for molecular breeding strategies in *O. fragrans*.

## 1. Introduction

Plasma membrane intrinsic proteins (PIPs) constitute a significant subfamily within the aquaporin (AQP) family, playing a pivotal role in the maintenance of fluid balance, which is essential for organismal survival across diverse environmental conditions [1]. Since the discovery of aquaporin-1 (AQP1) in mammals in the late 20th century, genome and transcriptome sequencing projects have identified thousands of orthologous channels in archaeal, bacterial, and eukaryotic organisms [2]. PIPs, a type of aquaporin found in most plasma membranes of plant tissues and organs, play a crucial role in regulating water balance and physiological responses [3]. For instance, PIPs modulate water molecule movement within and across plant cells, thereby sustaining fluid homeostasis involved in the regulation of plant growth and development [4]. PIPs can be associated with biotic and especially abiotic stresses, such as drought, salinity, or tolerance to soils rich in heavy metals [1,5,6]. This multifaceted role of PIPs underscores their integral contribution to plant growth and developmental regulation and adaptation.

Flower opening is a vital physiological process for plant successful pollination and reproduction. The process of flower opening is mainly due to the expansion of petal cells [7]. A number of *PIP* genes involved in the petal expansion and flower opening have been identified in many flowering plants [8]. The *RhPIP2;1* gene has been reported to promote flower petal expansion and thus induce the flower opening. This process is associated with ethylene-regulated cell expansion in petals [9,10]. Meanwhile, *RhPIP1;1* may modulate flower opening under drought stress conditions [11]. Additionally, *PIPs* play a critical role in regulating the water balance and turgor pressure of plant cells, which directly impacts flower corolla development. In *Arabidopsis, PIP1;1* affects corolla size by modulating the water balance in corolla cells, with *PIP1;1* mutants displaying a pronounced reduction in corolla size [12]. In rice, *PIP2;1* also significantly influences corolla size, as its expression level is directly related to the turgor pressure of the corolla [13]. In maize, *PIP2;5* is involved in the regulation of water content in corolla cells. Overexpression of *ZmPIP2;5* results in a substantial increase in corolla size, whereas its knockout leads to a reduction in corolla size, indicating that this protein has a significant impact on corolla dimensions [14]. In other species, such as *Dianthus caryophyllus*, *Eucommia ulmoides*, *and Populus przewalskii*, most pieces of evidence have demonstrated that *PIPs* play important roles in regulating the flower opening process [12,15,16]. Although it has been known that *PIPs* play an important role in flower opening, the molecular mechanisms are largely unknown, especially in the woody ornamental species.

Drought is a major abiotic stressor that affects plant survival and production worldwide, as well as influencing plant flowering. PIPs are also integral to plant adaptations to drought stress, mediating a spectrum of physiological responses [17,18]. Overexpression of *MaPIP1;1* in banana improved drought tolerance, and the transcription factors such as *MaERFs*, *MaDREBs*, *MaMYBs*, and *MabZIPs* enhance drought tolerance in transgenic plants by directly binding to the *MaPIP1;1* promoter [19]. Similarly, overexpressing *SlTIP2;2* showed that the tolerance of salt and water stresses was improved in the transgenic tomato plants under field conditions [20]. Moreover, the accumulation of *NtPIP1;1* and *NtPIP2;1* transcripts was significantly decreased under drought stress. When the *NtPIP1;1* and *NtPIP2;1* co-expression formed a heterotetramer, heteromerization of NtPIP1;1 and NtPIP2;1 significantly enhanced water transport activity and improved drought tolerance [21]. Overall, PIP members are important for the plant’s drought tolerance and the recovery from the water-deficient condition. Elucidating the intricate mechanisms of PIPs’ functions can offer profound insights into the regulatory networks of plants and pave the way for innovative approaches to bolster plant performance and stress adaptability.

*Osmanthus fragrans*, a traditional Chinese ornamental plant, is renowned for its valuable fragrance, making it a popular choice in gardening and food applications. The flowers, essential for both their esthetic appeal and fragrance, significantly influence the overall appeal of *O. fragrans* [22]. However, environmental factors such as drought limit its distribution and affect flower opening. To investigate the molecular functions of *PIP* genes in *O. fragrans*, we conducted experiments involving the overexpression of the *OfPIP2* gene in *Nicotiana tabacum* (tobacco) and *Petunia* species. Our results indicated that *OfPIP2* not only increased the size of the flower crown but also enhanced drought tolerance in these plants. Additionally, we demonstrated that OfMYB28 acts as a positive regulator by binding to the *OfPIP2* promoter to modulate its expression in *O. fragrans*. In summary, our findings highlight the crucial role of the *OfPIP2* gene in regulating flower opening and enhancing resistance to drought stresses in *O. fragrans*. Our results offered valuable insights that could contribute to future efforts in improving the desirable traits of *O. fragrans* through molecular breeding strategies.

## 2. Results

### 2.1. OfPIP2 Expression and Subcellular Localization Analysis

**Figure 1:**
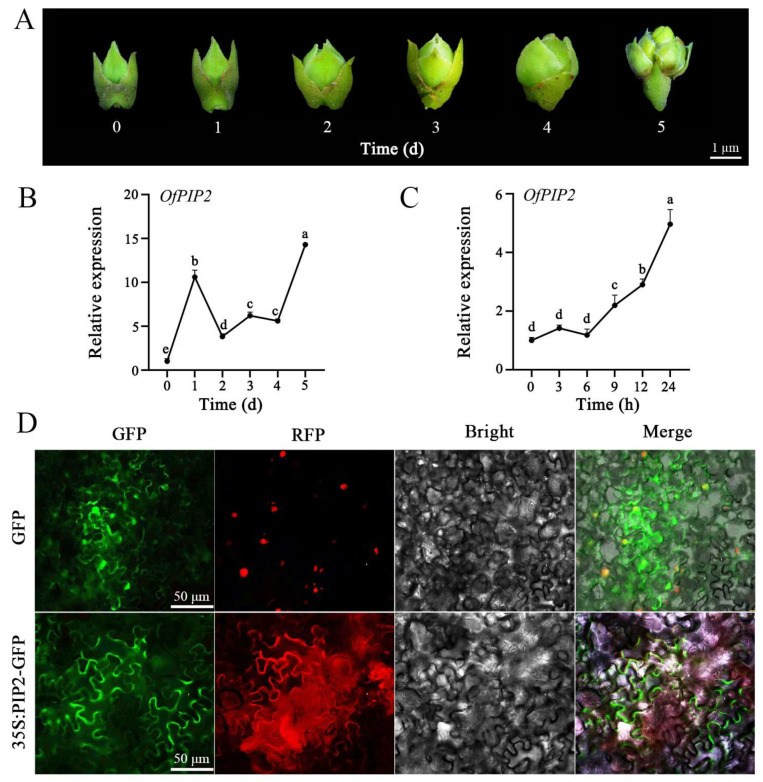
*OfPIP2* expression characteristics and subcellular localization in *O. fragrans.* (**A**) Phenotypic changes in flower buds. (**B**,**C**) Expression pattern of OfPIP2 during flower opening (**B**) and drought stress (**C**). The statistical analysis was performed using a one-way analysis of variance (ANOVA) followed by Duncan’s multiple range test (DMRT) with three biological replicates. *p*-values < 0.05 were considered significant, indicated by different letters. (**D**) Subcellular localization of *OfPIP2*. GFP: Green Fluorescence Channel; RFP: Red Fluorescence Channel; Bright Field: Bright Field Image; Merge: GFP, RFP, and Bright Field Merged Image; Scale bar = 50 µm.

The qRT-PCR analysis showed an overall increasing trend in *OfPIP2* expression throughout the flowering process in *O. fragrans* (Figure 1A,B). Meanwhile, *OfPIP2* expression was significantly upregulated during 24 h under drought stress treatment (Figure 1C). The expression results indicated that *OfPIP2* is involved in the regulation of flower opening and drought stress responses in *O. fragrans*. Additionally, the subcellular localization revealed that *OfPIP2* is located on the cell membrane (Figure 1D).

### 2.2. Promoter Analysis and Gus Staining

**Figure 2:**
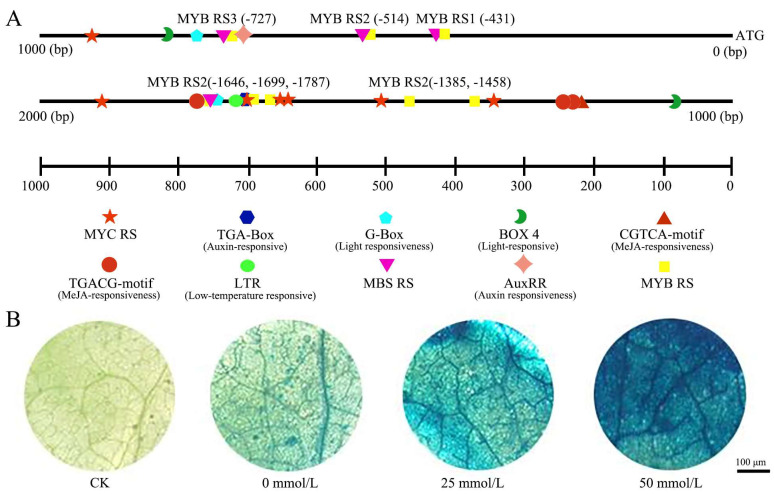
GUS staining analysis of the *OfPIP2* promoter. (**A**) The structure of the *OfPIP2* promoter. (**B**) The expression patterns of GUS driven by the *OfPIP2* promoter under different concentrations of mannitol treatment in transgenic tobacco leaves.

The promoter of the *OfPIP2* gene was analyzed and identified as having the presence of 16 distinct cis-acting elements (Figure 2A). This diverse array includes 11 MYB recognition and binding sites, 4 drought-responsive MYB binding sites, 2 sites that respond to the phytohormone methyl jasmonate, 2 elements associated with growth hormone responses, 5 light-sensitive elements, and 7 MYC response elements implicated in plant stress responses. To ascertain the OfPIP2 promoter’s responsiveness to drought stress, a series of histochemical GUS staining assays were conducted under varying concentrations of mannitol treatment (Figure 2B). The assays demonstrated a marked induction of GUS activity with increasing mannitol concentrations, indicating a direct correlation between mannitol-induced osmotic stress and promoter activity. The staining intensity escalated with enhanced mannitol concentrations, providing evidence for the promoter’s sensitivity to drought stress.

### 2.3. OfPIP2 Regulates Corolla Size in Petunia and Tobacco

**Figure 3:**
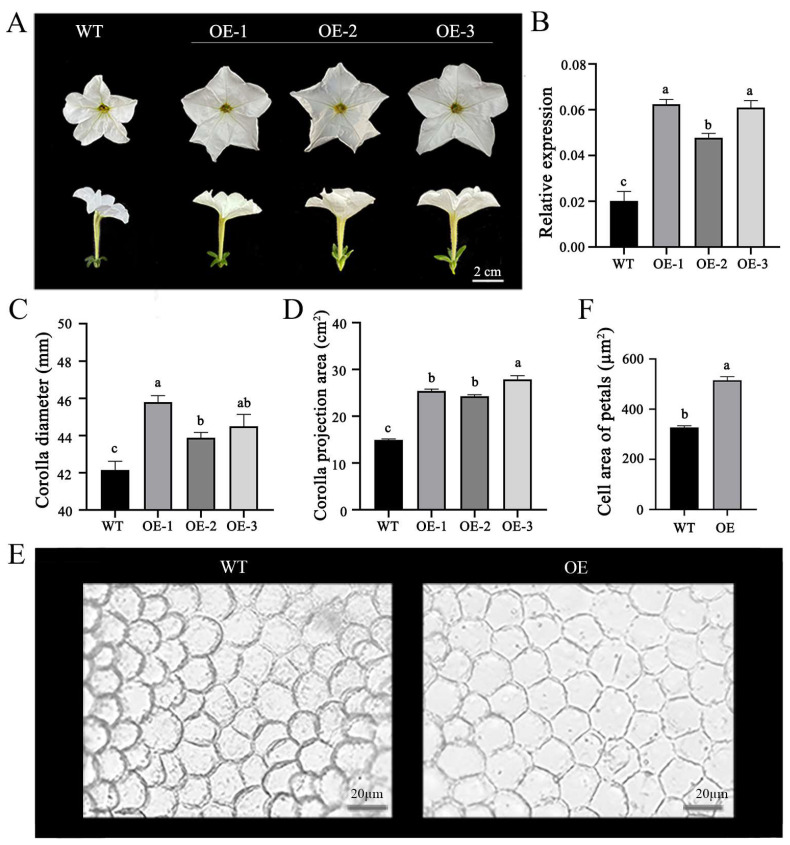
Phenotypic analysis of *OfPIP2* overexpression in *Petunia.* (**A**) Corolla and corolla tube. (**B**) Relative expression of *OfPIP2*-overexoressed transgenic lines and controls. (**C**) Flower corolla diameter. (**D**) Corolla projection area statistics. (**E**) Petal epidermal cells. (**F**) Petal epidermal cell area statistics. For (**B**,**C**,**E**), different lowercase letters represent significance between wt and overexpression lines by one-way ANOVA method (*p* < 0.05).

To elucidate the influence of the OfPIP2 gene on floral phenotypes, we ectopically expressed OfPIP2 in *Petunia* and tobacco plants. Our findings indicate that the overexpression of *OfPIP2* substantially enlarged the corolla size in both species. In transgenic *Petunia* lines overexpressing *OfPIP2*, the flowers demonstrated an increased corolla diameter and pronounced inward-curling margins relative to the WT plants (Figure 3A). The mean corolla diameter in the transgenic lines was augmented to 45.80, 43.88, and 44.50 mm in the OE-1, OE-2, and OE-3 lines, respectively, from a WT measurement of 42.14 mm (Figure 3C). The corolla projection area was significantly amplified in the transgenic lines to 24.60, 23.34, and 29.12 cm^2^ in OE-1, OE-2, and OE-3 lines, respectively, compared to the WT area of 15.74 cm^2^ (Figure 3D). Moreover, the petal epidermal cell area in the overexpression lines was markedly expanded to 515.14 µm^2^, surpassing the WT measurement of 327.24 µm^2^ (Figure 3E,F).

**Figure 4:**
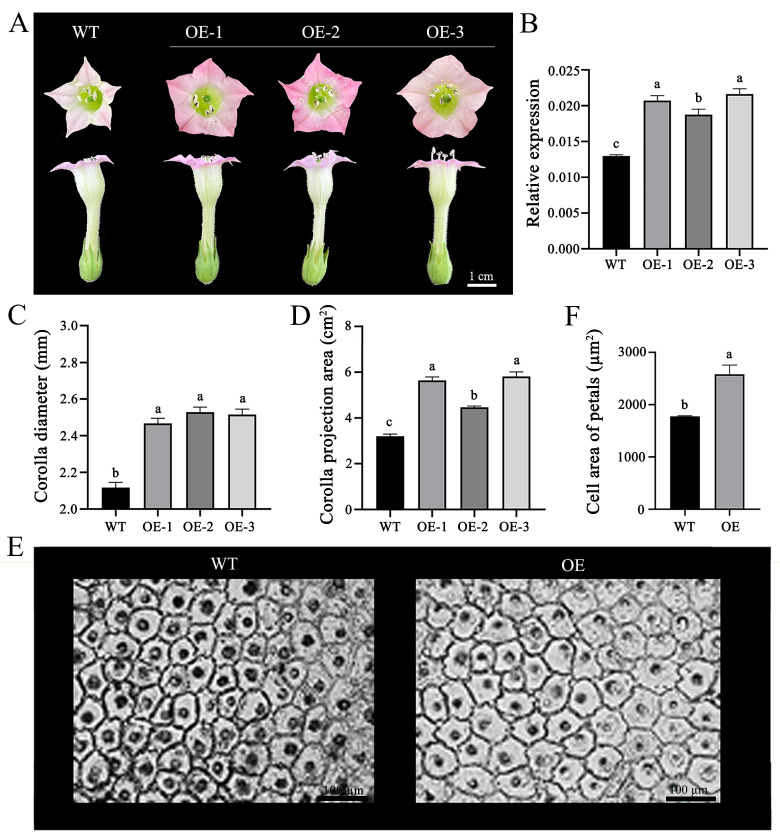
Phenotypic observation of *OfPIP2* transgenic tobacco petals. (**A**) Corolla and corolla tube. (**B**) Relative expression of *OfPIP2*-overexoressed transgenic lines and controls. (**C**) Flower corolla diameter statistics. (**D**) Corolla projection area statistics. (**E**) Epidermal cell area of petals. (**F**) Petal epidermal cell area statistics. For (**B**,**C**,**E**), different lowercase letters represent significance between wt and overexpression lines by one-way ANOVA method (*p* < 0.05).

In parallel, to ascertain the phenotypic modifications induced by *OfPIP2*, the gene was introduced into tobacco plants. The petal diameter in the overexpressed tobacco lines was correspondingly enlarged, reaching 45.80, 43.88, and 44.50 mm in the transgenic lines versus 42.14 mm in the WT (Figure 4A,C). The corolla projection area in the transgenic tobacco lines was also increased to 5.20, 4.54, and 5.84 cm^2^ in the OE-1, OE-2, and OE-3 lines, respectively, from a WT value of 3.40 cm^2^ (Figure 4D). Additionally, the petal epidermal cell area in the overexpressing lines was significantly larger, measuring 2480.56 µm^2^, in contrast to the WT area of 1780.43 µm^2^ (Figure 4E,F).

### 2.4. OfPIP2 Involved in the Regulation of Drought Tolerance in Petunia and Tobacco

**Figure 5:**
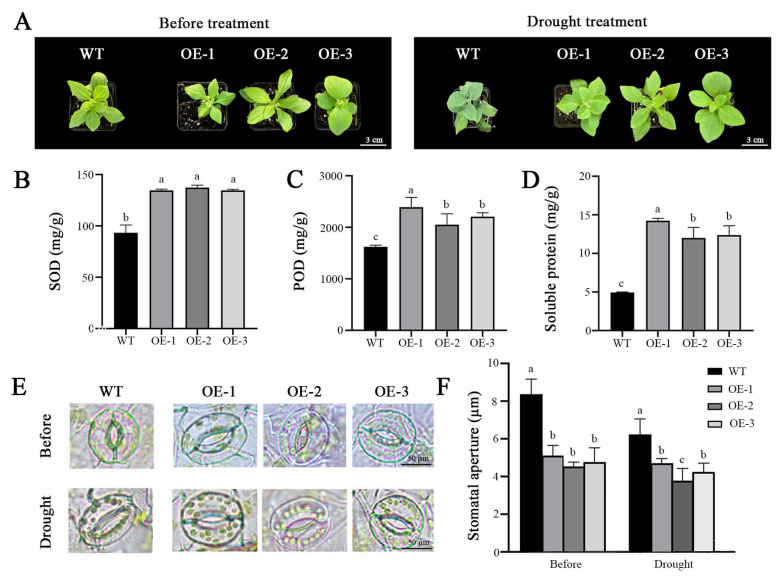
Overexpression of the *OfPIP2* gene improves drought tolerance under natural drought stress in *Petunia.* (**A**) Phenotypes of the wild-type and transgenic *Petunia* under natural drought for 15 days. (**B**–**D**) Physiological data statistics of superoxide dismutase content (SOD) (**B**), peroxidase content (POD) (**C**,**D**) soluble protein content. (**E**) The stomatal observation. (**F**) The stomatal conductance statistics. For (**B**–**D**,**F**), different lowercase letters represent significance between wt and overexpression lines by one-way ANOVA method (*p* < 0.05).

**Figure 6:**
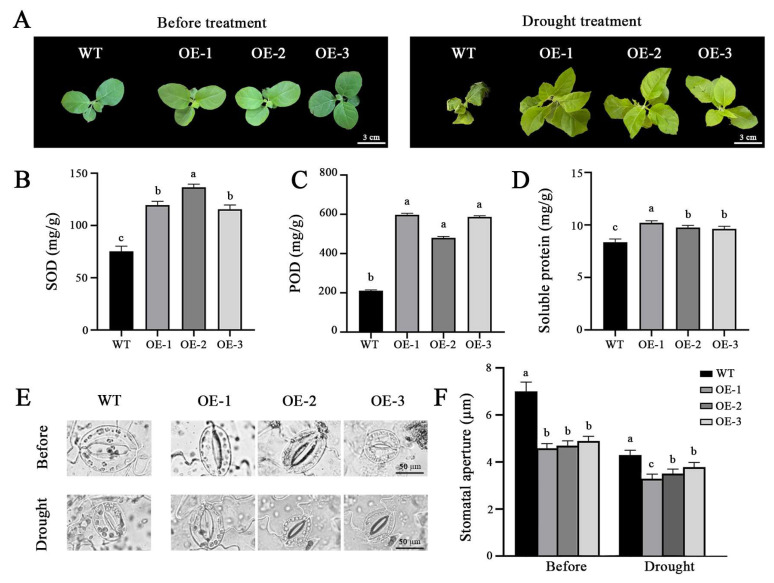
Heterologous expression of the *OfPIP2* gene improves drought tolerance under natural drought stress in tobacco. (**A**) The phenotypes of the wild-type and transgenic tobacco plants under natural drought treatment for 30 days. Physiological data statistics of (**B**) superoxide dismutase content (SOD), (**C**) peroxidase content (POD), and (**D**) soluble protein content. (**E**) The stomatal observation. (**F**) Stomatal conductance statistics. For (**B**–**D**,**F**), different lowercase letters represent significance between wt and overexpression lines by one-way ANOVA method (*p* < 0.05).

To ascertain the role of the *OfPIP2* gene in drought stress response, both transgenic *Petunia* and tobacco plants were subjected to a 15-day and 30-day natural drought treatment, respectively. Both transgenic plants demonstrated resilience, maintaining firm leaf texture after stress treatment, whereas the WT plants exhibited pronounced leaf drooping and wilting (Figure 5A and Figure 6A). In terms of physiological indicators, the *OfPIP2*-OE plants had elevated levels of POD, SOD, and soluble proteins relative to the WT plants after drought treatment (Figure 5B–D and Figure 6B–D). In addition, we further investigated the changes in stomatal conductance in both transgenic lines and found that the stomatal conductance in *OfPIP2*-OE plants had notably smaller stomatal apertures compared to the WT plants before stress treatment. Upon drought stress, both *OfPIP2*-OE and WT plants showed a substantial reduction in stomatal aperture, with the *OfPIP2*-OE plants showing a more significant decrease (Figure 5E,F and Figure 6E,F). These results suggest that the *OfPIP2* gene can improve the drought tolerance by reducing the stomatal conductance.

### 2.5. Transcription Factor OfMYB28 Positively Regulates OfPIP2 Expression

**Figure 7:**
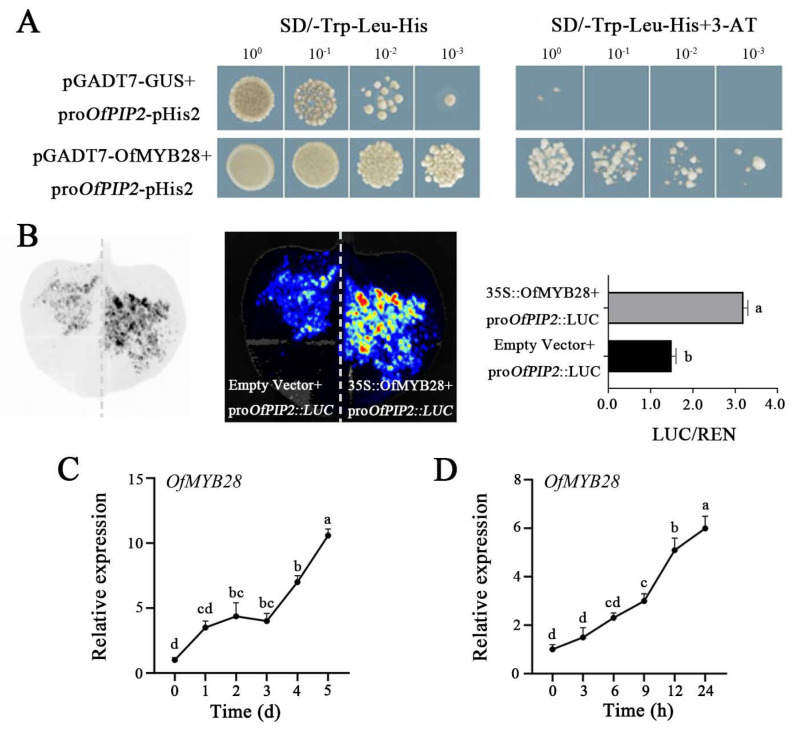
OfMYB28 potentially regulates *OfPIP2* expression. (**A**) OfMYB28 binds to the *OfPIP2* promoter by Y1H. (**B**) Dual-LUC analysis of *OfPIP2*. (**C**) Expression analysis of *OfMYB28* during flower opening. (**D**) Expression analysis of *OfMYB28* under drought treatment; the statistical analysis was performed using Duncan’s multiple range test (DMRT) with three biological replicates. Different lowercase letters represent significance between wt and overexpression lines by one-way ANOVA method (*p* < 0.05).

The transcription factor *OfMYB28* was initially identified through the yeast one-hybrid (Y1H) screening using the *OfPIP2* promoter. Then, we used the Y1H and Dual-LUC methods to confirm this interaction. When co-transformed with pGADT7-*OfMYB28* and *OfPIP2*-promoter-*pHis2* into yeast strain Y187, we observed successful growth of yeast on SD/-Trp-Leu-His medium (Figure 7A). Subsequently, the Dual-LUC assay demonstrated that OfMYB28 effectively activates the *OfPIP2* promoter (Figure 7B), resulting in a 2.13-fold increase in the LUC/REN ratio compared to the empty vector (Figure 7B). Additionally, the *OfMYB28* expression levels were examined during flower opening and under drought stress treatment in *O. fragrans*. We found that the expression of *OfMYB28* significantly increased during flower opening and after 24 h of drought stress treatment (Figure 7C,D). These findings suggest that OfMYB28 is able to enhance the expression of the *OfPIP2* gene by directly binding to its promoter, contributing to the flower opening and drought tolerance in *O. fragrans*.

## 3. Discussion

PIPs, integral to the aquaporin family, have garnered considerable research attention for their pivotal roles in water and solute transport processes [23]. Beyond their established function in water permeability regulation, PIPs are implicated in a spectrum of biological processes such as cell motility, secretion, and nutrient uptake [24]. Recent studies underscore their indispensable part in water balance maintenance, facilitation of plant cell growth and development, and amelioration of stress tolerance [25]. In *Arabidopsis*, drought stress has been shown to induce the expression of water channel proteins like *AtPIP1;4* and *AtPIP2;5* in leaves [26]. Although the functions of PIPs in model plants are well documented, there is a dearth of research on PIPs in *O. fragrans*. This study identified a plasma membrane aquaporin gene, *OfPIP2*, in which a significant upregulation was observed during the flower opening process and subsequent to drought treatment in *O. fragrans* (Figure 1). Meanwhile, GUS staining assays of the *OfPIP2* promoter demonstrated that staining intensity increased progressively with higher concentrations of mannitol, indicating that the *OfPIP2* promoter effectively drives the expression of the downstream GUS reporter genes (Figure 2). These results suggest a potential involvement of *OfPIP2* in the regulation of petal expansion and drought tolerance in *O. fragrans*.

### 3.1. OfPIP2 Involves in the Regulation of the Petal Size in O. fragrans

The flowering process involves irreversible growth and expansion of the petals, leading to the opening of the flower [27]. In most plant species, it is reported that the PIP family contributed to the petal expansion to regulate plant flowering. In rose (*Rosa hybrida* ‘Samantha’), *Rh-PIP2;1* is predominantly found in petal epidermal cells, with its expression significantly correlating to petal growth. Silencing *Rh-PIP2;1* mimics the ethylene-induced inhibition of petal expansion [28]. In barley, *HvPIP2;1* was identified to be abundantly expressed in lodicules and significantly upregulated in response to the flowering process, suggesting the importance of *HvPIP2;1* in the flowering process of barley [8]. To further validate the role of *OfPIP2* in the regulation of the flowering process, we performed transformations into *Petunia* and tobacco. We found that the *OfPIP2* overexpression significantly increased the petal diameter and corolla projection area of transgenic *Petunia* and tobacco; the cell area of the adaxial and abaxial surfaces in the *OfPIP2* overexpression lines was noticeably larger than those in the WT plant (Figure 3 and Figure 4). The results strongly support the hypothesis that *OfPIP2* might be a key plasma membrane aquaporin gene, which specifically influences the flowering process by the expansion of petal cells in *O. fragrans*.

### 3.2. OfPIP2 Enhanced the Drought Tolerance in O. fragrans

The impoverished environment drought condition is a major impactor, which limited the *O. fragrans* distribution [29]. Drought stress also affected the flowering processes, such as flower bud development, petal expansion, and flower opening [7]. In *O. fragrans*, overexpression of the *OfPIP2* gene can increase the survival rate of the transgenic *Petunia* and tobacco lines after the 15-day natural drought stress treatment, suggesting *OfPIP2* overexpression enhanced the drought tolerance in the transgenic lines (Figure 5 and Figure 6). These results are consistent with the other plant species, like banana [19], tomato [20]. Plants rely on stomata to facilitate gas exchange with their environment, which is critical for photosynthesis, water evaporation, and thermoregulation. The proper functioning of stomata is essential for these processes (McAdam et al. 2023) [30]. Under drought stress, stomatal conductance often decreases, which is closely linked to the expression of water channel protein genes [31]. This reduction in stomatal conductance limits water movement into and out of the plant, affecting both photosynthesis and transpiration. To mitigate water loss, plants employ strategies such as reducing stomatal aperture to conserve internal water [32]. Although stomatal closure is generally a passive process, during rehydration, stomata transiently open due to active osmotic adjustments between guard cells and epidermal cells [33]. In *O. fragrans*, stomatal observations revealed that *OfPIP2* overexpression induced stomatal conductance by adjusting the size and density of guard cells. This ability to maintain normal physiological activities under stress suggests that *OfPIP2* plays a significant role in enhancing drought tolerance through its regulatory functions. Furthermore, *AtPIP2;8* has been identified as a regulator that can inhibit stomatal closure, which can reduce drought tolerance, while PLATZ4 influences drought tolerance by regulating *AtPIP2;8* [34]. 

### 3.3. MYB28 Positively Regulated the OfPIP2 Expression

**Figure 8:**
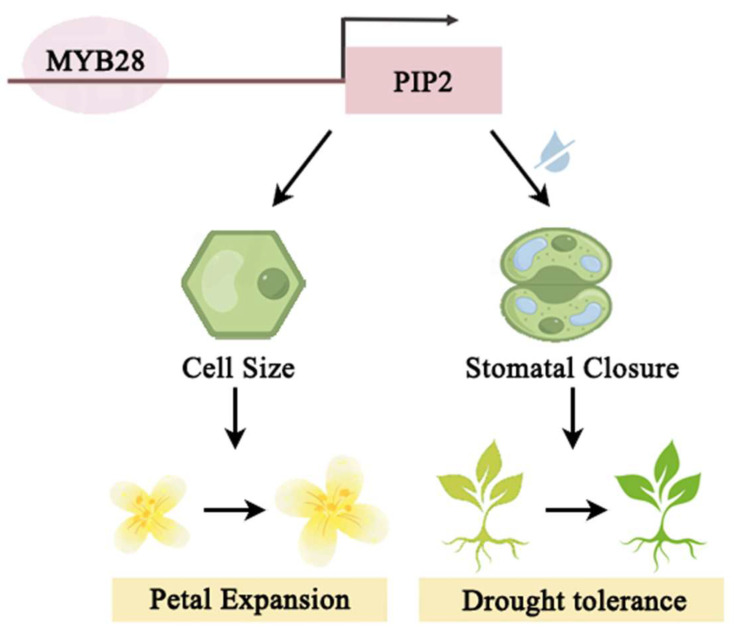
A proposed model for *PIP2*-mediated petal expansion and drought resistance in *Osmanthus fragrans*.

MYB transcription factors are integral in regulating plant growth and responses to abiotic stresses. A membrane-tethered MYB-like transcription factor, RhPTM, interacts with the aquaporins RhPIP2;1 from *Rosa hybrida*, and the phosphorylation of RhPIP2;1 promotes translocation of RhPTM into the nucleus in response to drought stress [35]. Similarly, the *MsPIP2;1* protein acts as a positive regulator, with its phosphorylation status under water stress influencing interactions with MYB transcription factors and modulating water tolerance in *Medicago sativa* [36]. These interactions highlight the complex regulatory networks involving MYB transcription factors and water channel proteins in enhancing plant resilience to water stress. However, there is limited information about how the MYB transcription factor promotes the *PIP* gene expression. In our study, we screened a MYB transcription factor, *OfMYB28*, which can directly bind the *OfPIP* promoter to positively regulate the expression utilizing Y1H and Dual-LUC (Figure 7). Furthermore, the expression analysis showed an overall increasing trend of *OfMYB28* during the flowering process and under drought stress treatment. The expression trend is consistent with the *OfPIP* expression in *O. fragrans*. All the results implied that the function of *OfPIP2* is involved in the regulation of petal size and drought tolerance, which is positively regulated by the *OfMYB28* transcription factor in *O. fragrans*, as illustrated in Figure 8. 

## 4. Materials and Methods

### 4.1. Growth Conditions and Treatments of O. frangrans 

The uniform *O. fragrans* cultivar ’Yanhonggui’ was maintained at Zhejiang Agriculture and Forestry University. All the materials were grown in pots at a temperature of 20 °C with a 12-h photoperiod and a relative humidity level of 60% in a greenhouse. According to Shiwei Zhong et al. [37], flower buds were collected at 0, 1, 2, 3, 4, and 5 days, respectively. For the drought stress treatments, the uniform 20-centimeter-long branches of *O. fragrans* ’Yanhonggui’ were selected and treated with 200 mmol/L D-mannitol according to the methods of Bin Dong et al. [29]. All the materials were maintained in a climatic chamber with a 12-h photoperiod at 25 °C and a relative humidity of 60%. Then, leaf samples were collected at 0, 3, 6, 9, 12, and 24 h after treatment and subsequently stored at −80 °C. All the experiments were conducted with three biological replicates.

### 4.2. RNA Extraction and First Strand cDNA Synthesis

Total RNA was extracted using the RNSprep Pure Kit (TianGen Biotech Co., Ltd., Beijing, China) according to the manufacturer’s instructions. First-strand cDNA synthesis was performed using ToloScript All-in-One RT EasyMix for qPCR (Tolobio, Shanghai, China).

### 4.3. Quantitative Real-Time PCR (qRT-PCR)

The qRT-PCR primers were designed using NCBI Primer-BLAST (http://www.ncbi.nlm.nih.gov, accessed on 1 July 2023), and the sequences are provided in Table S1. The *OfACT* gene served as the internal normalization control [38]. Each 10 μL qRT-PCR reaction mixture consisted of 5 μL SYBR Premix Ex Taq, 2 μL cDNA, 0.4 μL forward primer, 0.4 μL reverse primer, and 2.2 μL ddH_2_O. qRT-PCR was conducted using the LightCycler 480 II System (Roche, Basel, Switzerland). Each assay was performed with three biological replicates. The relative expression levels of the target genes were determined using the 2^−^^∆∆CT^ method [39].

### 4.4. Subcellular Localization

The sequence of ORF (open reading frame), with the stop codon removed, was amplified and then ligated into the *Nhe I* and *Xho I* sites of the pORE-R4 vector [40]. This construct was fused with green fluorescent protein (GFP) at the C-terminus to generate the *OfPIP2*-GFP plasmid. After confirming the construct by sequencing, the *OfPIP2*-GFP plasmid was transformed into the *Agrobacterium tumefaciens* strain GV3101 (Weidi Biotechnology Co., Ltd., Shanghai, China). Both the empty vector and the *OfPIP2*-GFP plasmid were transiently expressed in *Nicotiana benthamiana* leaves, with each experiment conducted in triplicate. GFP signals were detected and captured using a Zeiss LSM 710 confocal microscope (Carl Zeiss, Jena, Germany).

### 4.5. Promoter Analysis and Histochemical GUS Staining

The promoter sequence of the *OfPIP2* gene was obtained through genomic analysis. Cis-acting regulatory elements were identified using the PLANTCARE program (http://www.plantcare.co.uk, accessed on 1 July 2023) and Tbtools software v1.09 [41]. After analysis, the pCAMBIA1300GUS-*OfPIP2* fusion vector was constructed and transformed into *A. tumefaciens* strain GV3101. The transformed *Agrobacterium*, carrying either the empty vector or the pCAMBIA1300GUS-*OfPIP2* plasmid, was used to infiltrate the abaxial surface of *N. benthamiana* leaves. The infiltrated plants were incubated in the dark at 28 °C for one day and then transferred to light conditions. Afterward, the leaves were excised and placed in an MS solution containing 0, 25, or 50 mmol/L mannitol, and incubated for 3 h at 28 °C. Following incubation, both the transiently transformed leaves and control leaves (treated with different concentrations of mannitol) were subjected to GUS histochemical staining using the GUS staining kit (Coolaber, Beijing, China). After 24 h, the stained leaves were decolorized in 75% anhydrous ethanol. The ethanol was continuously replaced until the green color completely faded to white. Staining was observed under a microscope (Stemi 305, Zeiss, Germany). Three independent biological replicates were performed.

### 4.6. Transformation and Phenotype Investigation in Petunia and Tobacco

The entire coding sequence of *OfPIP2*, excluding the stop codon, was inserted into the pORE-R4-35AA vector using primers containing *NheI* and *XhoI* restriction sites, resulting in the construction of the fusion expression vector R4-OfPIP2. This vector was then used to transform *Agrobacterium tumefaciens* strain EHA105, which was cultured on lysogeny broth (LB) plates supplemented with 50 μg/mL kanamycin and 50 μg/mL rifampicin. The *Petunia hybrida* cv. ‘Mitchell Diploid’ was used to generate transgenic plants via the leaf–disk transformation method [42,43]. The tobacco cultivar Nicotiana tabacum L. ‘NC89’ was used for genetic transformation, following the method described by Sparkes et al. [44]. Leaf DNA was extracted to identify positive transgenic seedlings. Both transgenic and wild-type (WT) plants were used for phenotypic measurements, performed with vernier calipers. The experiment was repeated three times.

### 4.7. Physiological Index Measurement after Drought Treatment

To further assess the drought tolerance, 4-week-old *OfPIP2*-overexpressing transgenic *N. tabacum* and *Petunia hybrida* plants were evenly irrigated to ensure uniform growth, followed by natural drought treatments for 30 days in *N. tabacum* and 15 days in *Petunia hybrida* without external watering. All the plants were placed in a climatic chamber set at 25 °C with a 16-h light/8-h dark cycle to ensure that drought was the only stress factor. Then, the plant tissue (0.15 g) from treated transgenic plants was macerated in phosphate buffer (5 mL) and the volume adjusted to 10 mL under refrigerated conditions (4 °C). The homogenate was centrifuged, and the supernatant, enriched with crude enzymatic content, was isolated and refrigerated for subsequent analysis. Using the physiological marker assay kit (RUIXIN Biotech, Quanzhou, China), the physiological indexes, including malondialdehyde (MDA), superoxide dismutase (SOD), and peroxidase (POD), were measured to evaluate the drought tolerance of *OfPIP2*-overexpressing transgenic plants. Three independent biological replicates were performed.

### 4.8. Yeast One Hybrid (Y1H) Assay

The ORF of the *OfMYB28* gene was cloned into the pGADT7 vector at the *Nde I* and *Xho I* restriction sites. Concurrently, a 1500 bp fragment of the *OfPIP2* promoter sequence was ligated into the pHis2 vector. The recombinant plasmids, pGADT7-*OfMYB28* and pHis2-*ProOfPIP2*, were subsequently co-transformed into the yeast strain Y187 using the Coolaber Yeast Transformation Kit. To ensure the validity of our experimental outcomes, control transformations were conducted employing the empty vector pGADT7-*GUS*. Post-transformation, yeast colonies were assayed via spot tests on synthetic dropout medium (SD) lacking tryptophan, leucine, and histidine, further supplemented with the competitive inhibitor 3-amino-triazole (3-AT) at a final concentration of 20 mmol/L to enhance the stringency of selection. The plates were incubated under standardized conditions at 30 °C for a duration of 3 days to allow for subsequent analysis. 

### 4.9. Dual-Luciferase (Dual-LUC) Assays

The full-length cDNA of *OfMYB28* was cloned into the pORE-*R4* vector, and the promoter fragment of *OfPIP2* was inserted into the pGreenII0800-LUC vector. The resulting recombinant constructs were then transformed into *A. tumefaciens* strain GV3101 containing the pSoup helper plasmid. After incubating at 28 °C for 3 days, bacterial colonies were collected and resuspended in a solution containing 10 mM MES, 10 mM MgCl_2_·6H_2_O, and 200 µM acetosyringone (AS) to achieve an optical density OD_600_ of 1.0. Subsequently, the bacterial cultures were mixed in equal volumes, corresponding to the different protein-promoter combinations, and infiltrated into *N. benthamiana* leaves. After 48 h, the third or fourth leaf from each plant was harvested for Dual-LUC assays, following the manufacturer’s protocol (Promega, Durham, NC, USA).

### 4.10. Statistical Analysis

Data significance analysis was performed using Excel 2010 and SPSS Statistics 22, with Duncan’s multiple range test applied for statistical comparison (* *p* < 0.05, ** *p* < 0.01). Graphical representations of the data were created using GraphPad Prism 8.0.

## 5. Conclusions

In summary, a plasma membrane aquaporin gene *OfPIP2* was identified in *O. fragrans*, which effectively enhanced the corolla size and drought tolerance of *Petunia* and *tobacco* plants. Meanwhile, a transcription factor, OfMYB28, as a key regulator, directly binds to the *OfPIP2* promoter, inducing *OfPIP2* expression. These results illuminate the molecular mechanisms of the OfMYB28-*OfPIP2* module underlying flower opening and stress tolerance in *O. fragrans*, which might serve as a key player in orchestrating the trade-off between petal growth and drought resistance in *O. fragrans*.

## Data Availability

The original contributions presented in this study are included with the article/Supplementary Material; further inquiries can be directed to the corresponding authors.

## Supplementary Materials

The following supporting information can be downloaded at: https://www.mdpi.com/article/10.3390/ijms251910716/s1.
