# Supplementary material for: A Plasma Membrane Intrinsic Protein Gene OfPIP2 Involved in Promoting Petal Expansion and Drought Resistance in Osmanthus fragrans
Source: Int J Mol Sci. 2024 Oct 5;25(19):10716. doi: 10.3390/ijms251910716 (PMC11477222; doi:10.3390/ijms251910716)
Supplement: Supplementary file 1 [file ijms-25-10716-s001.zip › ijms-3239933-supplementary.pdf]

Table S1 Information of Primer sequences

	Gene Name	Primer Sequences
Relative expression (qRT-PCR)	*OfPIP2*	F: CTTGGCGAGGAAGGTGTCTC<br>R: TGCCGATAATCTCAGCACCG
	OfMYB28	F: AACTCATGGCAAGGAATCTCTCT<br>R: CCTGATCCATTTGGGCAGTTG
	OfACT	F: CCCAAGGCAAACAGAGAAAAAAT<br>R: ACCCCATCACCAGAATCAAGAA
Subcellular Localization	*pORE-R4-OfPIP2*	F: TTCTTCACTGTTGATAGCTAGCATGACAAAGGAAGTTAGCGAAGAAG<br>R: AGGCCTTCTAGAAAAACTCGAGGTTGGTGGGGTTGCTCCTG
GUS staining	*pCAMBIA1300GUS-OfPIP2*	F: TGTAAAACGACGGCCAGTGGCCTGTCATGATTAAAAGCTGT<br>R: AACCAAGAAAATGAAGGAGAAACTTCGCTCTAGCTTTCTCTCTCTAAA
Dual-LUC assay	*Pro-OfPIP2::GUS*	F: CAGGAACCAGGGCGTATCTCGGCCTGTCATGATTAAAAGCTGT<br>R: TAATACGACTCACTATAGGGCTTCGCTCTAGCTTTCTCTCTCTAAA
	pORE-R4-OfMYB28	F: TTCTTCACTGTTGATAGCTAGCATGGGCCATGGCCACCAT<br>R: AGGCCTTCTAGAAAAACTCGAGGGGATTGATCCAGCCTGAAGG
Yeast one-hybrid	*pHIS2-proOfPIP2*	F: GACTCACTATAGGGC GAATTCATGACAAAGGAAGTTAGCGAAGAAG<br>R: CCAGGAATTTCTAGA CCGCGGTTAGTTGGTGGGGTTGCTCCT
	pGADT7-OfMYB28	F: CATATGGCCATGGAGGCCAGTGAATTCATGGGCCATGGCCACCAT<br>R: CATCTGCAGCTCGAGCTCGATGGATCCCTAGGGATTGATCCAGCCTGAA

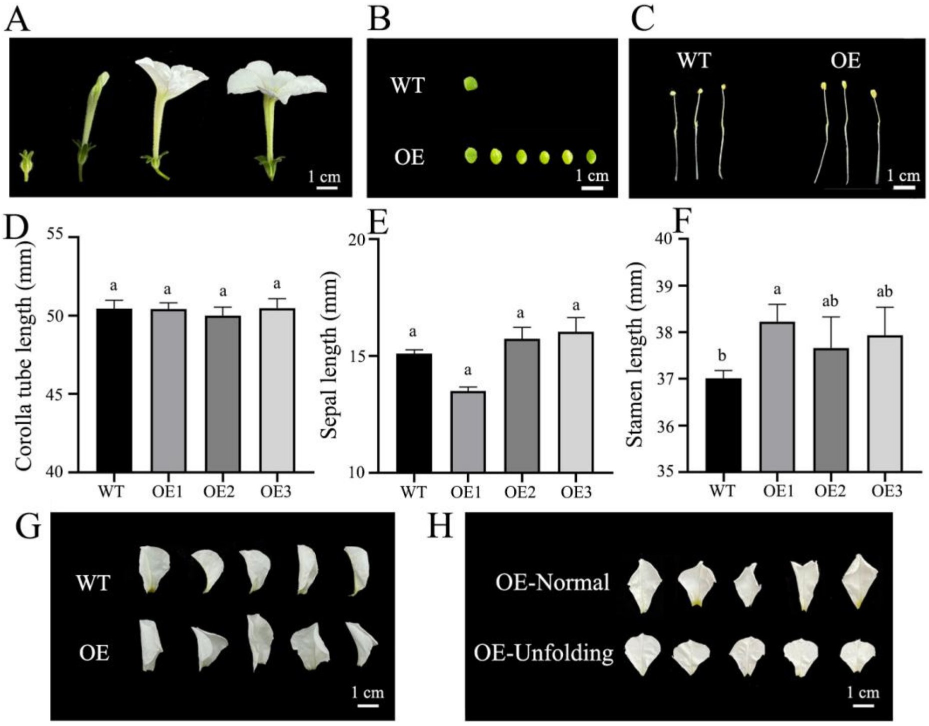

Figure S1. Phenotypic analysis of *OfPIP2* overexpression in *Petunia.*

A. Petunia flower opening process. B. Sepal. C. Stamen. D. Corolla tube length Statistics. E. Speal length Statistics. F. Stamen length Statistics. G. Width of petals. H. Normal and unfolded in contrast. Different lowercase letters indicate significant differences between WT and overexpression lines, determined by one-way ANOVA ($p < 0.05$).

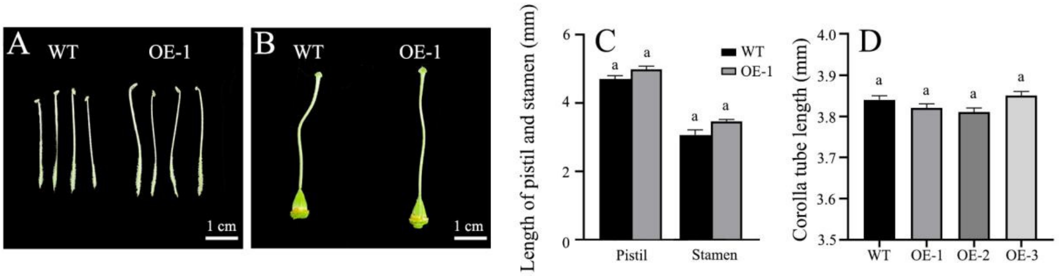

Figure S2. Phenotypic analysis of *OfPIP2* overexpression in tobacco.

A. Stamen. B. Pistil. C. Stamen and pistil length Statistics. D. Corolla tube length Statistics. Lowercase letter indicate significant differences between WT and overexpression lines, determined by one-way ANOVA ($p < 0.05$).
